# Comparison of microbial detection rates in microbial culture methods versus next-generation sequencing in patients with prosthetic joint infection: a systematic review and meta-analysis

**DOI:** 10.1186/s13018-023-03973-5

**Published:** 2023-08-16

**Authors:** Hideo Kato, Mao Hagihara, Nobuhiro Asai, Takumi Umemura, Jun Hirai, Yuka Yamagishi, Takuya Iwamoto, Hiroshige Mikamo

**Affiliations:** 1 Department of Clinical Infectious Diseases, Aichi Medical University, 1-1, Yazakokarimata, Nagakute, Aichi 480-1195 Japan; 2 Department of Pharmacy, Mie University Hospital, Mie, Japan; 3 Division of Clinical Medical Science, Department of Clinical Pharmaceutics, Mie University Graduate School of Medicine, Mie, Japan; 4 Department of Molecular Epidemiology and Biomedical Sciences, Aichi Medical University Hospital, Aichi, Japan

**Keywords:** Meta-analysis, Next-generation sequencing, Culture, Prosthetic joint infection

## Abstract

**Background:**

Accurate diagnosis of prosthetic joint infection (PJI) enables early and effective treatment. However, there is currently no gold standard test for microbial detection of PJI and traditional synovial fluid culture is relatively insensitive. Recently, it has been reported that sonicating fluid culture and next-generation sequencing (NGS) improve microbial detection rates. Hence, we performed a systematic review and meta-analysis to compare microbial detection rates in microbial culture methods with and without sonication versus NGS.

**Methods:**

We systematically searched EMBASE, PubMed, Scopus, CINAHL, and Ichushi databases and other sources (previous reviews) until August 2022. We evaluated the detection rates of pathogens in NGS and microbial cultures using samples of synovial or sonicated fluid.

**Results:**

Of the 170 citations identified for screening, nine studies were included. Pooled analysis indicated that NGS had the highest detection rate among the microbial detection methods (NGS vs. sonicated, odds ratios [OR] 5.09, 95% confidential interval [CI] 1.67–15.50; NGS vs. synovial, OR 4.52, 95% CI 2.86–7.16). Sonicated fluid culture showed a higher detection rate than synovial fluid culture (OR 2.11, 95% CI 1.23–3.62).

**Conclusion:**

NGS might be useful as a screening tool for culture-negative patients. In clinical settings, sonicated fluid culture is a practical method for diagnosing PJI.

## Background

Prosthetic joint infections (PJI) are critical complications, which accounts for 25% of failed knee arthroplasties and 15% of failed hip arthroplasties [1]. PJI is associated with a 5-year mortality rate higher than that of common malignancies [2]. Moreover, the cost of treating PJI is substantial, estimated at over a billion dollars in 2017 [3]. Considering that the prevalence of arthroplasties is projected to twice by 2030, the cost of PJI in the healthcare budgets cannot be ignored [3]. Therefore, PJI has adverse effects on quality of life and cost to no small degree.

PJI with unknown causative pathogens is typically treated with a broad-spectrum antibiotic regimen to treat many pathogens related with PJI. Therefore, the key to successful treatment is early and accurate microbial diagnosis, as the use of effective antibiotics is one of important parts for the treatment of PJI.

Despite the publication of clinical guidelines and definitions for PJI, there are still many challenges in its diagnosis [4–6]. Currently, there is no gold standard test for microbial detection of PJI and it has been reported that 40% of culture-negative patients meet the clinical diagnostic criteria for PJI [7]. Traditional synovial fluid culture can be insensitive, and 7–12% of the pathogens in PJI are not detected in even cases that multiple cultures are utilized with extended culture time [8]. Therefore, it is important to address this issue for improving the detection rate of pathogens that can cause PJI.

Recently, several microbial detection methods have been proposed to overcome the challenges in diagnosing PJI. Sonicated fluid culture has been revealed to increase microbial yield by disrupting the bacterial biofilm in PJI [9]. Moreover, sonicated samples showed higher sensitivity and specificity than synovial fluid samples [10]. Second, next-generation sequencing (NGS) has shown high value in the diagnosis of pathogens of various infectious diseases. NGS has been reported to increase microbial detection rates in tuberculous meningitis and lung infections [11, 12]. In patients with PJI, NGS identified the same pathogens in 82.9% of the culture-positive ceases and detected the pathogens in 84.0% of the culture-negative cases [13]. Thus, NGS is a tool to identify a wide range of PJI pathogens. However, use of NGS in a clinical practice is still controversial.

To date, previous studies including meta-analyses have shown the clinical usability of NGS in identifying causative pathogens of PJI [14, 15]. However, in these meta-analyses, the accuracy of NGS was evaluated, while the detection rate between microbial culture methods and NGS was not compared. Moreover, these meta-analyses included the studies with same durations and patients with non-infectious aseptic failure. Hence, we compared the microbial detection rates between microbial culture methods and NGS in patients who diagnosed as PJI using a systematic review and meta-analysis. The purpose of this study was to reveal a strategy in diagnosis of PJI. This might allow a better selection of the most appropriate microbial culture to be used for the detection of pathogens in patients with PJI.

## Materials and methods

This analysis was performed and reported in accordance to the PRISMA guidelines except for protocol registration [16, 17]. The institutional review board approval was exempted in this study.

### Study design and data sources

Comprehensive literature review searches of EMBASE, PubMed, CINAHL, Scopus, and Ichushi databases and other sources (previous reviews [14, 15]) until August 26, 2022, using the following terms: (“prosthetic joint infection” OR “periprosthetic joint infection” AND (“next-generation sequencing”) AND (“culture”). Language was restricted to English and Japanese. Additional searches were conducted by analyzing the references from the retrieved papers and reviews.

### Study selection

Two investigators (HK and MH) screened the titles and abstracts of the articles. The full-text articles were then reviewed to apply the inclusion criteria, and the articles for the final qualitative synthesis and meta-analysis were identified. The third author (HM) resolved any disagreements through discussions. Studies that met the following criteria were extracted: (i) randomized controlled trials (RCT), retrospective observational, or cohort studies; (ii) patients with PJI; and (iii) studies that investigated the microbial detection rates in NGS and microbial cultures with samples of synovial or sonicate fluid of the hip or knee. PJI was diagnosed according to the Infectious Diseases Society of America (IDSA) or Musculoskeletal Infection Society (MSIS) criteria [4]. Studies were excluded based on the following criteria: (i) Irrelevant reviews, letters, personal opinions, book chapters, and meeting abstracts; (ii) insufficient data regarding detection rates; and (iii) NGS and microbial culture methods were not studied. No restriction was placed on the NGS and microbial culture methods. Moreover, the study with the largest number of included patients among publications with duplicate data was selected.

### Data collection and risk-of-bias assessment

The following data were independently manually extracted by two study investigators (HK and MH): study design, settings, study period, country of study, participants and sample size, sample types, NGS methodology, and culture. The outcome of interest was the detection rate of pathogens from samples of synovial or sonicated fluid of the hip or knee. According to previous study [18], the risk-of-bias was assessed independently by two reviewers (HK and MH) using the RoBANS tool [19]. The criteria for assessing the risk-of-bias included the selection of participants, confounding variables, measurement of exposure, blinding of outcome assessment, incomplete outcome data, and selective outcome reporting.

### Statistical analysis

According to a previous study [20], all extracted data were analyzed using Review Manager (RevMan, version 5.4; Nordic Cochrane Collaboration, Oxford, UK). The degree and proportion of statistical heterogeneity were evaluated using the chi-squared test and the I-squared (*I*^2^) measure, respectively. Heterogeneity was defined as significant when the P value was less than 0.1 or the *I*^2^ value was greater than 50%. Random effects models were applied to heterogeneous data, and fixed effects models were applied to homogenous data. Risk was calculated using odds ratios (OR) and 95% confidence interval (CI). The pooled OR and 95% CI were calculated using a fixed effects model and a random effects model, and OR from these results were compared.

## Results

### Systematic review

**Fig. 1:**
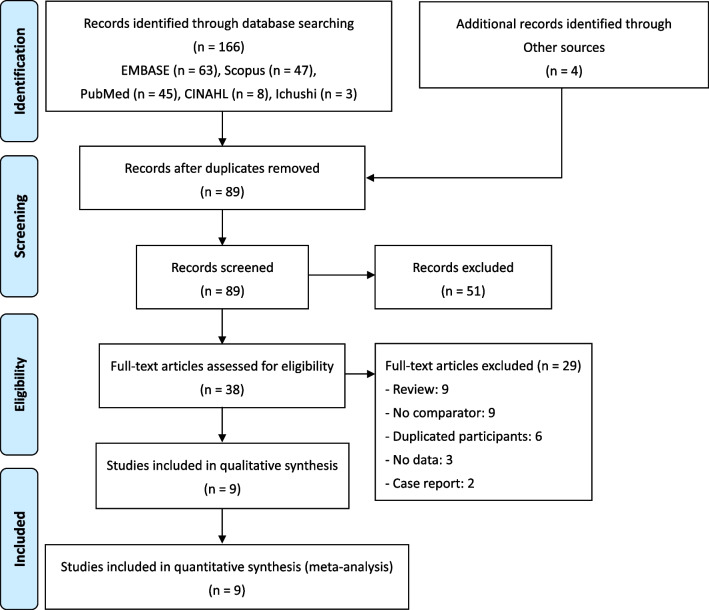
PRISMA flow diagram of the selection of eligible studies

The literature search resulted in a total of 170 potentially relevant screening studies. An additional relevant study was conducted using other sources. Thirty-eight articles were chosen for the full-text review, and nine studies were included in the systematic review and meta-analysis [13, 21–28]. Figure 1 shows the full list of reasons for exclusion. The characteristics of the nine studies are summarized in Table 1. Six were prospective studies [13, 21–24, 26], whereas the others were retrospective studies [25, 27, 28]. All studies were single-center. Five studies were conducted in US [13, 21, 22, 25, 26], and the others in the China [23, 24, 26, 27]. All samples were collected preoperatively or during surgery. For microbial cultures, six studies collected sonicated fluid samples [22–26, 28], and seven studies used synovial fluid samples [13, 21, 23–27]. For NGS, five studies collected sonicated fluid samples [13, 23, 25, 26, 28], and four studies used synovial fluid samples [21, 22, 24, 27]. All studies tested aerobic and anaerobic cultures as daily routine microbial culture conventionally. The aerobic and anaerobic cultures were incubated at 35–37 °C for 5 to 14 days and for 7–14 days (Table 1). Four studies reported positive predictive value of over 96% in NGS [23–26], while the others did not report it [13, 21, 22, 27, 28].

**Table 1:** Characteristics of the studies included in the meta-analysis

Study	Study design	Setting	Period	Country of study	Participants	Sample Size	Previous antibiotic	Sample types	NGS positive predictive value
Thoendel M, 2018	Prospective study	Single-center	2011–2016	US	Patients who underwent revision arthroplasties	*n* = 213	*n* = 131	Sonicated fluid	NR
Ivy M, 2018	Prospective Study	Single-center	Apr 1998–Jun 2018	US	Patients who underwent revision knee procedures	*n* = 107	*n* = 46	Synovial fluid	NR
Tarabichi M, 2018	Prospective Study	Single-center	Jun–Nov 2016	US	Patients who underwent revision arthroplasties	*n* = 28	*n* = 28	Synovial fluid	NR
Zhang C, 2019	Prospective Study	Single-center	Dec 2016–Dec 2018	China	Patients who underwent revision arthroplasties	*n* = 24	*n* = 15	Synovial fluid; Sonicated fluid; NGS, sonicated fluid	96%
Huang Z, 2020	Prospective Study	Single-center	Mar 2017–Jul 2018	China	Patients who underwent revision arthroplasties	*n* = 49	*n* = 18	Synovial fluid; sonicated fluid; NGS, synovial fluid	97.9%
Flurin L, 2021	Retrospective Study	Single-center	2007–2019	US	Patients who underwent revision of a total elbow arthroplasty	*n* = 47; synovial culture, *n* = 15	*n* = 19	Synovial fluid; sonicated fluid; NGS, sonicated fluid	98.0%
He R, 2021	Prospective Study	Single-center	Oct 2017–Apr 2019	China	Patients who underwent revision arthroplasties	*n* = 40	*n* = 9	Synovial fluid; sonicated fluid; NGS, sonicated fluid	94.7%
Yin H, 2021	Retrospective study	Single-center	Jul 2017–Dec 2019	China	Patients who underwent revision arthroplasties	*n* = 15	NR	Synovial fluid	88%
Hong HL, 2023	Retrospective study	Single-center	2011–2016	US	Patients who underwent resection arthroplasties	*n* = 208	*n* = 128	Sonicated fluid	NR

Study	Methodology	
	NGS	Culture
Thoendel M, 2018	Sonicated fluid samples performed microbial DNA enrichment using the MolYsis Basic5 kit (Molzym, Bremen, Germany). DNA extraction and whole genome amplification was performed using MoBio Bacteremia DNA isolation kits (Qiagen, Hilden, Germany) and the Qiagen REPLI-g Single Cell kit, respectively. Agencourt Ampure XP beads (Beckman Coulter, Brea, CA) and TE pH 8.0 (Integrated DNA Technologies, Coralville, IA) were used for purifying amplified DNA. Samples were sequenced with the Illumina HiSeq 2500 (Illumina, San Diego, CA)	Sonicate fluids were prepared from resected prosthetic hip and knee components using vortex and sonication methods previously described (aerobic and anaerobic cultures)
Ivy M, 2018	Synovial fluid samples performed microbial DNA enrichment using a MolYsis Basic5 kit (Molzym, Bremen, Germany). The pelleted bacteria were subjected to DNA extraction and isolation using a MoBio Bacteremia DNA isolation kit (Qiagen, Hilden, Germany). Whole-genome amplification was carried out using a Qiagen REPLI-g Single Cell kit (Qiagen) according to the manufacturer’s instructions. Amplified DNA was purified using Agencourt Ampure XP beads (Beckman Coulter, Brea, CA) according to the manufacturer’s protocol with elution in Tris–EDTA (TE) buffer, pH 8.0 (Integrated DNA Technologies, Coralville, IA)	Synovial fluid samples with a volume of over 1 ml was inoculated into a BD Bactec Peds Plus/F bottle and incubated for 5 d on a Bactec 9240 instrument prior to November 2012 and on a Bactec FX instrument (BD Diagnostic Systems, Sparks, MD) after November 2012. Synovial fluid samples with a volume of < 1 ml were put onto 5% sheep blood agar and BBL Chocolate II agar and into BBL fluid thioglycolate medium (BD Diagnostic Systems, Sparks, MD), which was followed by incubation at 35–37 °C for 5 d. In anaerobic culture, anaerobic Remel thioglycolate broth without indicator with vitamin K and hemin (Thermo Fisher Scientific, Lenexa, KS) was inoculated and incubated for 7–14 d, and BBL Centers for Disease Control and Prevention anaerobe 5% sheep blood agar (BD Diagnostic Systems) was inoculated and incubated anaerobically at 35–37 °C for 7–14 d
Tarabichi M, 2018	The DNA was amplified using PCR and sequenced on the Ion Torrent Personal Genome Machine system sequencing platform (Thermo Fisher Scientific). The generated sequences were read using the National Institutes of Health GenBank database	The routine cultures including aerobic and anaerobic bacterial cultures were performed
Zhang C, 2019	The DNA was extracted using the TIANamp Micro DNA Kit (DP316, Tiangen Biotech). DNA libraries were sequenced using the standard protocol of the BGISEQ-500 sequencing platform (BGITianjin, Tianjin, China). The reference genomes in the database were downloaded from the National Center for Biotechnology Information	Synovial and sonicated fluid were tested by Gram staining and acid-fast staining, followed by putting on blood agar plates. The remaining synovial and sonicated fluid were injected into BACTEC Peds Plus/F culture bottles (Becton Dickinson, Germany) and cultured in the BACTEC 9050 Culture System (Becton Dickinson, Germany). The cultures were incubated at 37 °C for 5 d (aerobic cultures) and 14 d (anaerobic cultures)
Huang Z, 2020	DNA libraries were sequenced using the BGISEQ-500 platform (BGI-Wuhan, Wuhan, China)	The implants were putting into 500 mL saline solution (Chimin Health Management, Taizhou, China), and the solutions spun for 30 s, followed by sonication for 3 min at 40 kHz (Woxing, Wuxi, China). Each 50 mL of sonicated fluid was concentrated to a final volume of 1 mL by centrifugation (1490×*g* for 10 min). The samples were cultivated for 6 d (aerobe) and at least 14 d (anaerobe)
Flurin L, 2021	All amplified samples underwent sequencing and were processed using a library preparation, normalization, and sequencing protocol from Illumina. The library (600 mL) was submitted to sequencing on an Illumina MiSeq with a 500 cycle V2 Nano kit (Illumina)	Culture results were collected through retrospective review of the electronic medical record (aerobic and anaerobic cultures)
He R, 2021	The processing the sample, nucleic acid extraction, construction of DNA libraries, sequencing and bioinformatic analysis were performed	Synovial and sonicated fluid were sent for routine testing including aerobic and anaerobic cultures and cultivated in a blood culture BD bottle on the BD- BACTEC-9120–9240 instrument (BD, Switzerland) up to 14 d
Yin H, 2021	DNA was extracted using the TIANamp Micro DNA Kit. The extracted DNA was quantified by Qubit 2.0, and then the samples were subjected to 20 M 50-bp single-end sequencing on the BGISEQ-50 platform. Sequencing data were classified by simultaneously aligning to four Microbial Genome Databases (NCBI)	The fluid was cultivated in aerobic, anaerobic, and blood culture bottles and cultivated for 14 d, respectively
Hong HL, 2023	DNA was extracted on a MagNA Pure 96 system. The extracted DNA was amplified by PCR on a LightCycler 480 II. Libraries were sequenced using a 2 × 250 V2 nano kit on an Illumina MiSeq	Samples were cultivated in an aerobic and an anaerobic sheep blood agar plate at 35 ℃ in 5–7% CO_2_ for 5 d (aerobe) and 14 d (anaerobe), respectively

*NGS* next-generation sequencing, *PCR* polymerase chain reaction, *PJI* prosthetic joint infection, *RAG* relative abundance in genus-level

The risk-of-bias assessment results are shown in Table 2. The risks-of-bias regarding the selection of participants, confounding variables, measurement of exposure, incomplete outcome data, and selective outcome reporting in all studies were low. Only one trial was blinded [19].

**Table 2:** Risk-of-bias in included studies

Study	Selection of participants	Confounding variables	Measurement of exposure	Building of outcome assessment	Incomplete outcome data	Selective outcome reporting
Ivy M, 2020	Low risk	Low risk	Low risk	High risk	Low risk	Low risk
Tarabichi M, 2018	Low risk	Low risk	Low risk	High risk	Low risk	Low risk
Thoendel M, 2018	Low risk	Low risk	Low risk	High risk	Low risk	Low risk
Zhang C, 2019	Low risk	Low risk	Low risk	High risk	Low risk	Low risk
Huang Z, 2020	Low risk	Low risk	Low risk	High risk	Low risk	Low risk
Flurin L, 2021	Low risk	Low risk	Low risk	Low risk	Low risk	Low risk
He R, 2021	Low risk	Low risk	Low risk	High risk	Low risk	Low risk
Yin H, 2021	Low risk	Low risk	Low risk	High risk	Low risk	Low risk
Hong HL, 2023	Low risk	Low risk	Low risk	High risk	Low risk	Low risk

### Meta-analysis

#### Microbial detection rates in sonicated fluid culture and NGS

**Fig. 2:**
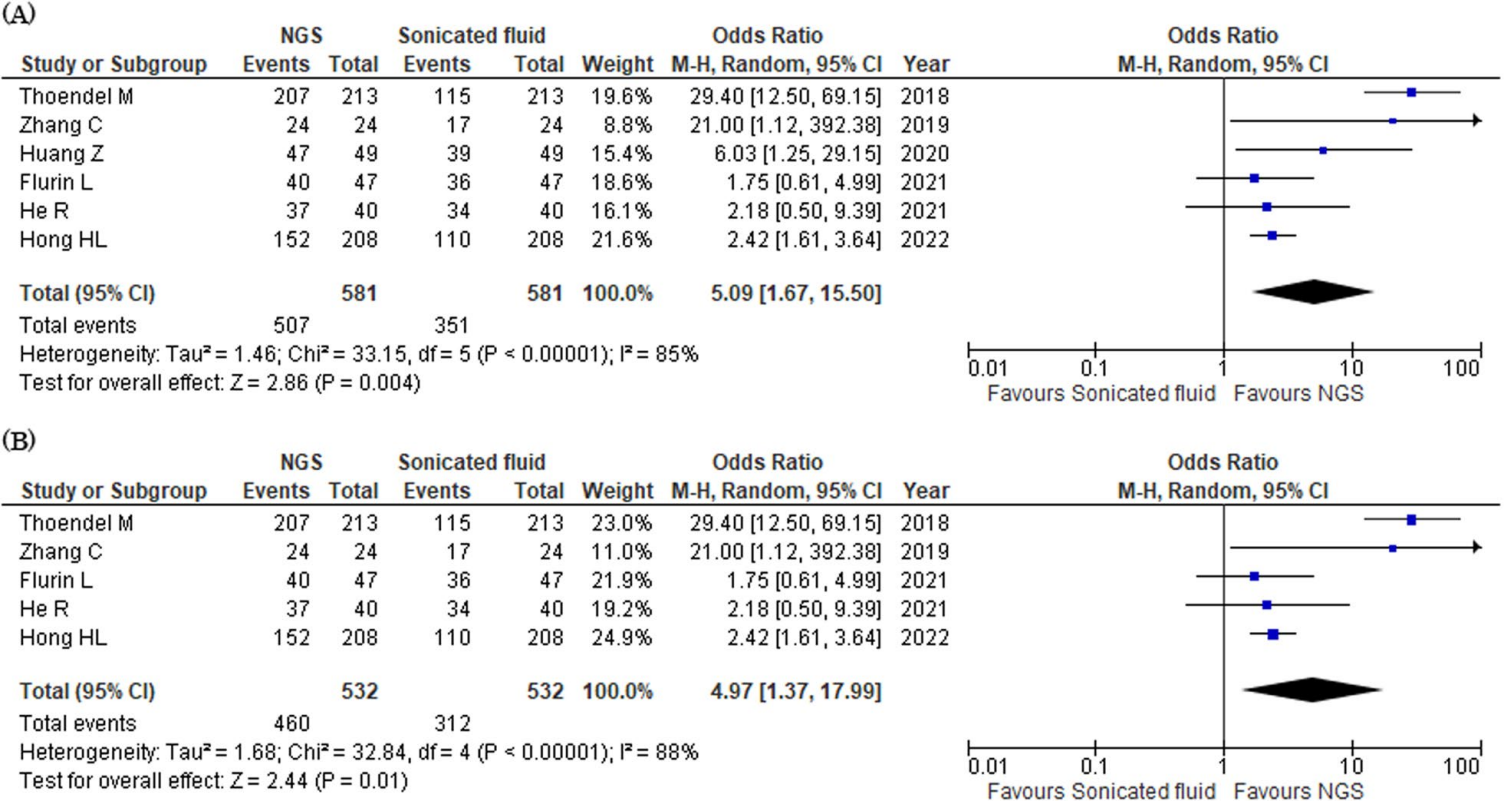
Forest plots of odds ratios for microbial detection rates in sonicated fluid culture versus NGS. **a** NGS of sonicated and synovial fluids. **b** NGS of sonicated fluid. *CI* confidential interval, *M–H* Mantel–Haenszel, *NGS* next-generation sequencing

Six studies reported microbial detection rates in sonicated fluid culture and NGS [22–26, 28]. Of the 581 patients, sonicated fluid culture and NGS groups detected microbials in 351 (60.4%) and 507 (87.3%), respectively. The detection rates in NGS resulted in a significantly higher pooled OR than that in sonicated fluid culture (OR 5.09, 95% CI 1.67–15.50, *I*^2^ = 85%, Fig. 2a). In five studies using sonicated fluid to NGS, the detection rate was significantly higher in NGS than in culture (OR 4.97, 95% CI 1.37–17.99, *I*^2^ = 88%, Fig. 2b).

#### Microbial detection rates in sonicated and synovial fluid cultures

**Fig. 3:**
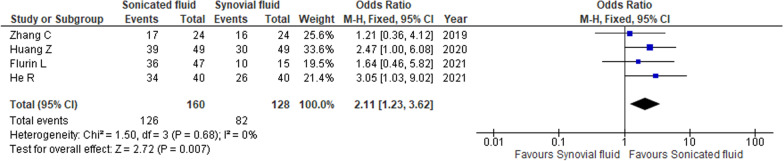
Forest plots of odds ratios for microbial detection rates in sonicate versus synovial fluid cultures. *CI* confidential interval, *M–H* Mantel–Haenszel

Four studies reported microbial detection rates in sonicated and synovial fluid cultures [23–26]. The detection rate in the sonicated fluid culture was 78.8%, while it was 64.1% in the synovial fluid culture. Sonicated fluid culture was associated with a significantly higher detection rate than the synovial fluid culture (OR 2.11, 95% CI 1.23–3.62, *I*^2^ = 0%, Fig. 3).

#### Microbial detection rates in NGS and synovial fluid culture

**Fig. 4:**
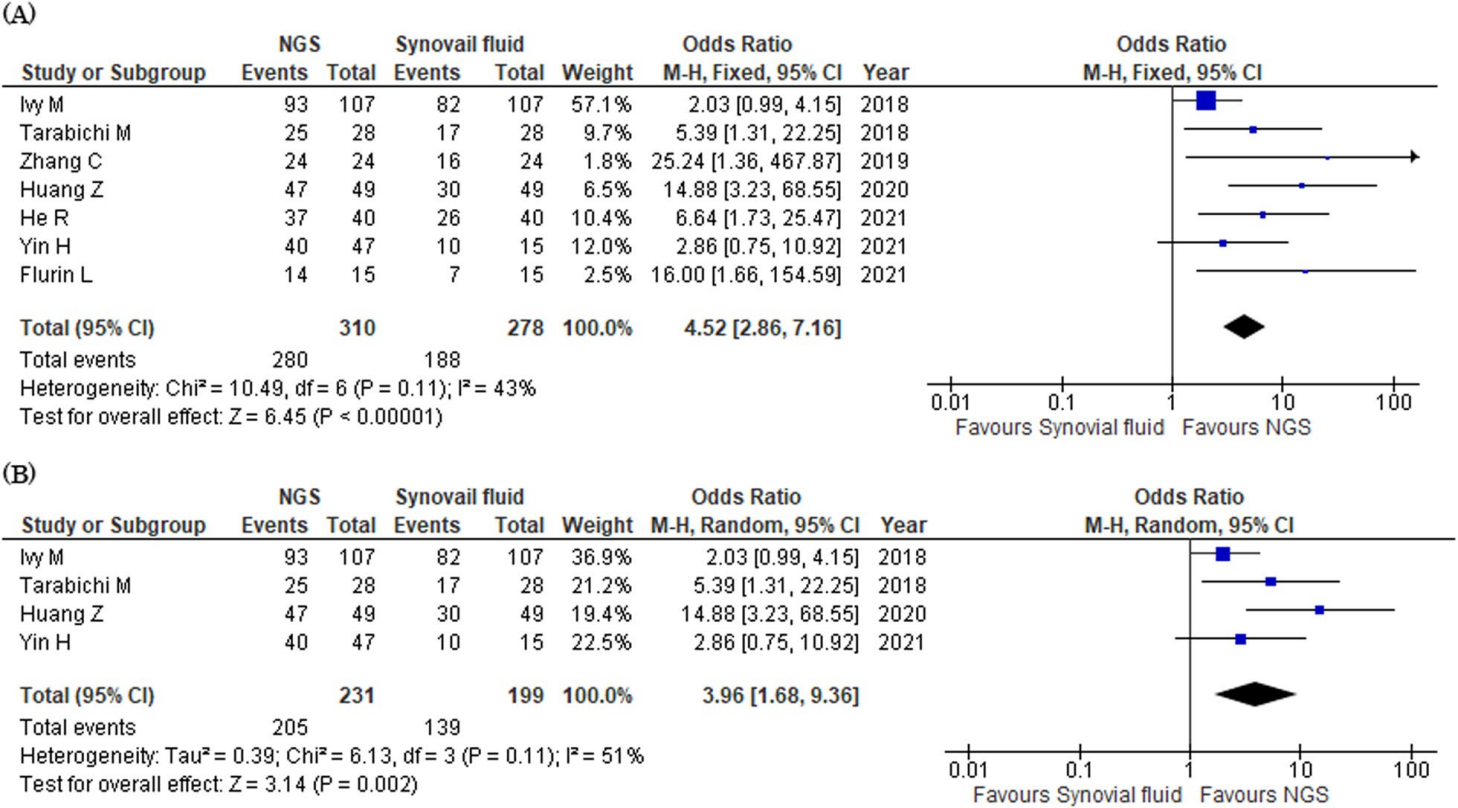
Forest plots of odds ratios for microbial detection rates in NGS versus synovial fluid culture. **a** NGS of sonicated and synovial fluids. **b** NGS of synovial fluid. *CI* confidential interval, *M–H* Mantel–Haenszel, *NGS* next-generation sequencing

Seven studies reported microbial detection rates in NGS and synovial fluid culture [13, 21, 23–27]. The pathogen was isolated using NGS and synovial fluid culture in 280 (90.3%) and 188 (67.6%) patients, respectively. The detection rate in NGS was significantly higher than that in synovial fluid culture (OR 4.52, 95% CI 2.86–7.16, *I*^2^ = 43%, Fig. 4a). In four studies using synovial fluid to NGS, the detection rate was significantly higher than in NGS than in culture (OR 3.96, 95% CI 1.68–9.36, *I*^2^ = 51%, Fig. 4b).

## Discussion

To date, this is the novel meta-analysis to compare the detection rates of NGS with those of microbial culture methods in patients with of PJI. This study compared microbial detection rates using NGS and synovial and sonicated fluid cultures in patients with PJI. Consequently, the detection rate was the highest in NGS, and sonicated fluid culture had a higher detection rate than synovial fluid culture.

Pathogens may be difficult to cultivate in patients receiving antibiotic treatment or that are detected with infections caused by fastidious microorganisms [29]. To overcome these limitations, microbial diagnosis of PJI has been improved by advances in culture methods such as sonication of samples [30]. In the present study, sonicated fluid culture was associated with a significantly higher detection rate of pathogens in patients with PJI than in synovial fluid culture. Therefore, the sonication can be used generally in clinical settings due to an easy and valuable technology and is also included in the diagnostic criteria of the European Bone and Joint Infection Society [31]. Moreover, the application of diagnostic methods using sonicated fluid has also been reported in specific populations. In general, the sensitivity of sonicated fluid cultures is 88%, while the percentage of positive cultures in sonicated fluids is 81% in patients who received antibiotics within 14 d before surgery, and the reduction in sensitivity is not recognized between patients with and without antibiotic therapy up to 14 d [32]. A previous study reported that the most frequent type was delayed infection when PJI was classified as early (< 3 months), delayed (3–12 or 24 months), or late-acute (occurring > 12–24 months after surgery) [33]. Among these, sonication has been reported to improve the etiological diagnosis of PJI in patients with delayed infection [33]. This has been explained by the evidence that the sonication may release microorganisms forming a biofilm from the surface of implant [34]. Therefore, sonicate fluid culture appears to be a useful clinical diagnostic method for the detection of pathogens in patients with PJI. However, the usefulness of NGS in the specific populations is unclear since in all the studies included in our meta-analysis reported no data regarding antibiotics use and infection types of PJI.

NGS generate thousands of individual sequences using a single broad-range polymerase chain reaction (PCR). This can provide exhaustive information about joint organisms and understand the joint microbiome. However, several organisms including opportunistic pathogens and contaminants have been detected in specimens from joint sites. Thus, NGS may be difficult to distinguish such bacteria from causative pathogens of infections. Therefore, the most concerning point is whether NGS can accurately guide the treatment. Our meta-analysis indicated that there was a correlation between NGS and the positive cultures. Recently, several studies have reported the clinical usability of NGS. The diagnostic sensitivity and specificity of NGS for PJI were 93% and 95%, respectively [14]. Moreover, it has been demonstrated that the species-level specificity of NGS is 88%, with a 95% CI of 77–94% [35]. Another study investigated whether patients ultimately benefited from NGS in diagnostic efficiency [36]. More patients adjusted antibiotics in the NGS group than in the culture methods group based on the pathogenic microbiology report (70.0% vs. 43.9%, *P* = 0.016), and more patients showed improved clinical symptoms in the early stage in the NGS group than in the culture methods group (60.6% vs. 37.9%, *P* = 0.032). Regarding mortality, the 28- and 90-d mortality rates were significantly lower in patients undergoing NGS testing than in those undergoing culture method test, with *P* values of 0.008 and 0.002, respectively [37]. Therefore, NGS may contribute appropriate treatment. However, the finding [37] targeted patients with pneumonia rather than those with PJI. Further studies are required to clarify its clinical utility in the treatment of PJI.

Information on causative pathogens that can be quickly provided during treatment is important for the administration of appropriate antibiotic agents. Indeed, the first results of NGS are provided within 24 h, which enables earlier implementation of appropriate treatment [38]. Moreover, the study comparing the detection rates in three microbial detection methods (synovial and sonicated cultures and NGS) concluded that NGS might replace microbial culture methods in patients with PJI [26]. However, NGS is currently not widely used in clinical practice. For the reasons that application of NGS is limited by the burden of sample preparation, expensive equipment, and high operating costs compared to culture methods, many hospitals and laboratories is difficult to be equipped with NGS. Moreover, there is still discussion on the way to interpret the results and clinical contexts that should be used. Recently, a study on the application of NGS to the diagnosis of PJI mentioned that the pre-test probability determined by the clinical picture and other laboratory investigations should be closely examined when interpreting the results of NGS [21]. Moreover, it has been reported that NGS is best suited to clinical cases where the pre-test probability of PJI is high [39]. Therefore, NGS seems to be more useful as a screening tool for the causative pathogens of PJI, especially in culture-negative patients. However, the development of low-cost NGS is needed in the future.

Our meta-analysis had some limitations, the most important of which was the lack of sufficient data. The number of studies included in our meta-analysis was small, and all the included studies were single-center studies. Therefore, our findings might increase the likelihood of reporting and selection bias. However, our meta-analysis included 713 patients more than previous studies (range, 15–213 patients). Moreover, there was no heterogeneity in our results, and one prospective study was included in our meta-analysis. Second, the methodology for each clinical microbial diagnosis could not be unified. Future studies should focus on the differences in methodology. Finally, the included studies did not mention whether same microbials were found if both tests detected microbials. However, the detected microbials were thought to be highly causative in patients diagnosed as PJI according to the IDSA or MSIS criteria [4, 18]. Further basic and clinical studies are necessary to resolve these issues.

## Conclusions

In conclusion, our meta-analysis revealed that NGS had the highest pathogen detection rate in patients with PJI and might be useful as a screening tool for culture-negative patients. Sonicated fluid culture showed a higher detection rate of pathogens in patients with PJI than synovial fluid culture. Given the current state in clinical settings, sonicated fluid culture is a practical method for diagnosing PJI.

## Data Availability

All relevant data are within the manuscript and its Supporting Information file.

## Abbreviations

CI: Confidential interval
NGS: Next-generation sequencing
OR: Odds ratios
PJI: Prosthetic joint infection
